# Trends in the Treatment and Survival of Pancreatic Cancer: Analysis of 23,339 Patients Diagnosed Between 2010 and 2017

**DOI:** 10.1002/cam4.71248

**Published:** 2025-09-19

**Authors:** Marko Damm, Miriam Heinig, Jonas Rosendahl, Patrick Michl, Ulrike Haug, Sebastian Krug

**Affiliations:** ^1^ Department of Internal Medicine I University Hospital Halle, Martin‐Luther‐University Halle‐Wittenberg Halle (Saale) Germany; ^2^ Department of Clinical Epidemiology Leibniz Institute for Prevention Research and Epidemiology – BIPS Bremen Germany; ^3^ Department of Internal Medicine IV University Hospital Heidelberg Heidelberg Germany; ^4^ Faculty of Human and Health Sciences University of Bremen Bremen Germany

**Keywords:** chemotherapy, epidemiology, pancreatic cancer, resection, survival

## Abstract

**Introduction:**

With a rising incidence and unchanged poor prognosis, pancreatic cancer is increasingly becoming a focus of gastroenterological oncology, but there is a lack of real‐world data. The aim of the current study was to investigate trends in survival and treatment patterns by analyzing German health claims data.

**Methods:**

Pancreatic cancer patients diagnosed between 2010 and 2017 were identified from the German Pharmacoepidemiological Research Database (GePaRD, approximately 20% of the German population). Data on demographics, tumor treatment within 1 year after diagnosis, and survival were extracted.

**Results:**

The study population comprised 23,339 patients with a median age of 74 years (IQR 66–80) and 44% with localized and 56% with metastatic disease. Overall, 52.4% received any chemotherapy, and curative intended resection was performed in 28.3%. Neoadjuvant and adjuvant therapy were performed in 4.4% and 58.7% of the cases, respectively. The median overall survival of the whole study population was 7.84 months. Patients diagnosed in the most recent period (2014–2017) had a significantly better prognosis (8.20 months (95% CI 7.97–8.43)) than patients who were diagnosed in the earlier period (2010–2013) (7.54 months (95% CI 7.31–7.70), *p* < 0.001), with an age‐, sex‐, and stage‐adjusted hazard ratio of 0.87 (95% CI 0.85–0.9). Over time, the most pronounced treatment trends have affected patients with localized disease, with increasing frequency of resection and neoadjuvant therapy and decreasing frequency of best supportive care.

**Conclusion:**

This comprehensive insight into survival and treatment of pancreatic cancer in Germany shows presumably medically beneficial therapy trends with, however, only marginal improvements in prognosis to date.

## Graphical abstract

This study, one of the largest European real‐world investigations, provides a comprehensive analysis of treatment patterns and survival outcomes in pancreatic cancer. While curative‐intent therapies, such as neoadjuvant therapy and tumor resection, have increased and survival has improved over time, the prognosis remains poor.

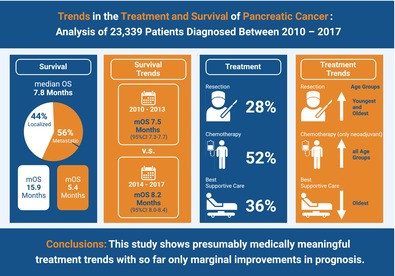

## 1 Introduction

Pancreatic ductal adenocarcinoma (PDAC) is an aggressive malignancy with a mortality‐to‐incidence ratio of approximately 95% [1]. Globally, the incidence has more than doubled in the last 30 years, currently estimated at over 500,000 cases per year. The highest incidence rate worldwide was documented for Western Europe with approximately 8.8/100,000 [2]. In Germany, the lifetime risk of developing pancreatic cancer is approximately 1.9% [3]. Based on the latest estimates, the incidence of pancreatic cancer will steadily increase in the Western world and will be one of the leading causes of cancer‐related mortality [4, 5]. The poor prognosis is characterized by a low rate of primary resectable PDAC (15%–20%) and a pronounced resistance to therapy in advanced stages [6].

Nevertheless, progress has also been achieved for patients with PDAC. Intensified adjuvant chemotherapy [7] and multimodal concepts for borderline [8] and locally advanced [9] pancreatic cancer have led to an increase in survival.

Given the epidemiological facts and the data from clinical studies, there is still a great need to evaluate the care of patients with PDAC in the real‐world setting. National registries and cohort studies from the Netherlands and Denmark demonstrate that most patients with PDAC are still being transferred to best supportive care. Although the number of patients with surgical interventions and/or systemic oncological therapy approaches has increased in the last decade, the median survival of all PDAC cases was low, at 4–5 months [10, 11].

In Germany, basic epidemiological data for PDAC are collected by cancer registries with full population coverage [3]. In addition to information on incidence and mortality (including relative 5‐year survival), the UICC stage and histology are also recorded. However, the extent to which the use of available therapeutic procedures has changed over the years in Germany cannot be determined based on currently available cancer registry data in Germany.

In the present study, we aimed to describe trends in cancer therapy and survival in a cohort of PDAC patients diagnosed between 2010 and 2017 based on German health claims data.

## 2 Methods

### 2.1 Data Source

We used the German Pharmacoepidemiological Research Database (GePaRD) which is based on claims data from four statutory health insurance providers in Germany and currently includes information on approximately 25 million individuals who have been insured with one of the participating providers since 2004 or later. In addition to demographic data, GePaRD contains information on drug dispensations as well as outpatient (i.e., from general practitioners and specialists) and inpatient services and diagnoses [12]. The German health care system is characterized by uniform access to all levels of care and free choice of providers.

### 2.2 Study Population and Study Design

For the valid identification of incident PDAC patients in German health claims data, we used an algorithm which was developed based on case reviews and indirectly validated by comparing incidence with cancer registry data. The algorithm also takes into account potential miscoding of pancreatic cancer due to pre‐existing other cancers that might have metastasized to the pancreas (Appendix S1). In this study, we included patients identified based on this algorithm and diagnosed between 2010 and 2017. Patients were excluded if they had received any treatment or diagnosis indicating neuroendocrine pancreatic neoplasms. Furthermore, patients with interruption of continuous insurance for more than 30 days during follow‐up were excluded. Patients were followed until death or end of observation on December 31, 2019, whichever occurred first.

### 2.3 Characterization of Included Patients

We described included patients regarding age, sex, stage at diagnosis, and treatment during the first year of follow‐up (i.e., the first year after diagnosis). Information on stage at diagnosis is not available in claims data but was estimated based on ICD codes indicating distant metastases, as previously described [13]. We differentiated between “localized” (no distant metastasis) and “metastasized” (distant metastasis) stage. Lymph node involvement tends to be underrecorded in this data source and was therefore not considered a separate category. Furthermore, there was no information available regarding the pathology results.

Regarding the description of treatment, we focused on initial therapy, i.e., cancer therapy (resection, chemotherapy, and radiotherapy) received in the year after diagnosis. Where applicable, we differentiated between neoadjuvant (prior to resection/explorative laparotomy if unresected) and adjuvant (at or after resection up until the end of the 1‐year time period after diagnosis) therapy. We also determined the time from diagnosis to resection (days) in resected patients. We used the following (not mutually exclusive) categories to describe therapy: (1) curative resection, (2) any chemotherapy and/or radiotherapy, (3) adjuvant therapy, i.e., chemotherapy and/or radiotherapy after resection, (4) neoadjuvant therapy, i.e., chemotherapy and/or radiotherapy prior to resection/explorative laparotomy if unresected, or (5) no tumor therapy, i.e., best supportive care (BSC).

### 2.4 Data Analysis

We determined proportions to describe frequencies. The distribution of continuous variables (age, time to resection) was described based on medians and interquartile ranges. The description was done for all included patients and stratified by age groups (< 60, 60–74, ≥ 75 years), sex (male, female), stage (localized, metastasized), calendar period of diagnosis (2010–2013, 2014–2017) and year of diagnosis. Pearson correlation coefficients were used to analyze the correlation of two continuous variables. To describe overall survival, we used Kaplan–Meier analysis. In addition, we performed Cox proportional hazards regression analysis in order to describe changes in overall survival between patients diagnosed from 2010 to 2013 and 2014 to 2017, adjusted for age at diagnosis, sex, and stage at diagnosis (in the categories described above). We also conducted such analyses to compare survival between diagnosis years in resected and unresected patients. All analyses were performed using SAS software (SAS Institute, version 9.4, NC, USA).

## 3 Results

### 3.1 Characteristics of the Study Population

Overall, we identified 27,192 patients with pancreatic cancer diagnosed between 2010 and 2017. Of these, 1247 patients with codes indicating pancreatic neuroendocrine neoplasms were excluded (e.g., specific chemotherapy). Furthermore, 2606 patients whose insurance period was interrupted during the follow‐up period (e.g., insurance switch) were excluded (Table S2).

Thus, 23,339 patients with PDAC were finally included in the present study. Information on patient characteristics and treatment for the whole study population stratified by sex, age, and stage is shown in Table 1. The median age at diagnosis was 74 years (IQR 66–80) and 53.3% (*n* = 12,439) of the patients were female. Regarding stage distribution, 44.4% (*n* = 10,374) patients had localized disease, and 55.6% (*n* = 12,965) had metastatic disease.

**TABLE 1:** Characteristics of included patients.

	All	Sex		Age			Stage	
		Male	Female	< 60 years	60–74 years	≥ 75 years	Localized	Metastatic
Overall number^a^	23,339 (100%)	10,900 (46.7%)	12,439 (53.3%)	3018 (12.9%)	9456 (40.5%)	10,865 (46.6%)	10,374 (44.4%)	12,965 (55.6%)
Age at diagnosis (median)	74 (66–80)	73 (65–78)	75 (67–81)	54 (50–57)	69 (65–72)	80 (77–85)	74 (66–81)	73 (66–79)
Resection	6604 (28.3%)	3263 (29.9%)	3341 (26.9%)	1190 (39.4%)	3235 (34.2%)	2179 (20.1%)	5233 (50.4%)	1371 (10.6%)
Time to resection from diagnosis, days (median)	60 (36–83)	60 (37–84)	61 (35–83)	62 (36–84)	60 (35–83)	60 (37–83)	61 (36–84)	59 (33–82)
Total pancreatectomy^b^	826 (12.5%)	427 (13.1%)	399 (11.9%)	148 (12.4%)	415 (12.8%)	263 (12.1%)	674 (12.9%)	152 (11.1%)
Partial: Distal^b^	1175 (17.8%)	550 (16.9%)	625 (18.7%)	251 (21.1%)	565 (17.5%)	359 (16.5%)	836 (16%)	339 (24.7%)
Partial: Whipple^b^	4407 (66.7%)	2182 (66.9%)	2225 (66.6%)	758 (63.7%)	2154 (66.6%)	1495 (68.6%)	3564 (68.1%)	843 (61.5%)
Other^b^, ^c^	196 (3%)	104 (3.2%)	92 (2.8%)	33 (2.8%)	101 (3.1%)	62 (2.8%)	159 (3%)	37 (2.7%)
Explorative laparotomy, but no resection	1246 (5.3%)	618 (5.7%)	628 (5%)	198 (6.6%)	602 (6.4%)	446 (4.1%)	450 (4.3%)	796 (6.1%)
Any chemotherapy	12,224 (52.4%)	6072 (55.7%)	6152 (49.5%)	2091 (69.3%)	6205 (65.6%)	3928 (36.2%)	4609 (44.4%)	7615 (58.7%)
Any radiotherapy	922 (4%)	481 (4.4%)	441 (3.5%)	217 (7.2%)	464 (4.9%)	241 (2.2%)	455 (4.4%)	467 (3.6%)
Neoadjuvant therapy^d^	342 (4.4%)	169 (4.4%)	173 (4.4%)	116 (8.4%)	163 (4.3%)	63 (2.4%)	181 (3.2%)	161 (7.4%)
Adjuvant therapy^e^	3879 (58.7%)	1924 (59.0%)	1955 (58.5%)	757 (63.6%)	2094 (64.7%)	1028 (47.2%)	2925 (55.9%)	954 (69.6%)
Best supportive care^f^	8349 (35.8%)	3463 (31.8%)	4886 (39.3%)	514 (17%)	2101 (22.2%)	5734 (52.8%)	3466 (33.4%)	4883 (37.7%)

*Note:* Shown are n (% of study population) or median (interquartile range).

^a^ Row percentages here referring to the entire study population (*n* = 23,339). In the remaining table, column percentages are shown.

^b^ Percentage are based on all patients with resections in the respective cohort.

^c^ Unspecified partial resection of several segments of the pancreas.

^d^ Chemo‐ or radiotherapy within the period of PDAC diagnosis to resection or exploration. % are based on patients who were resected or had exploratory laparotomy without resection.

^e^ Chemo‐ or radiotherapy after curative resection within the 1st year after diagnosis. % are based on patients who were resected.

^f^ No tumor therapy (resection, chemo‐, or radiotherapy) in the 1st year after diagnosis.

Overall, 52.4% (*n* = 12,224) received any chemotherapy, and in 4% (*n* = 922) of all patients, radiotherapy was performed within 1 year after diagnosis. Curative‐intended resection was performed in 28.3% (*n* = 6604) of all patients; this proportion was 50.4% and 10.6% in patients with localized and with metastatic disease, respectively. The median duration from diagnosis to resection was 60 days (Interquartile range (IQR) 36–83 days). Whipple's procedure was the most commonly performed operation (4407/6604, 66.7%), followed by distal (1175/6604, 17.8%) and total pancreatectomy (826/6604, 12.5%). Explorative laparotomy, where subsequent resection was apparently not feasible, was performed in 5.3% (*n* = 1246) of all patients. Among those with resection or explorative laparotomy, 4.4% (342/7850) received neoadjuvant therapy (i.e., chemo‐ and/or radiotherapy before the surgical procedure). Among those with resection, 58.7% (3879/6604) received adjuvant therapy (i.e., chemo‐ and/or radiotherapy after resection) within 1 year after diagnosis. The percentage of patients with no specific tumor‐directed treatment within 1 year after diagnosis (best supportive care) was 35.8% (8349/23,339). The percentage of patients who received best supportive care was 33% in those with localized stage and 38% in those with metastatic disease.

There were no relevant differences between female and male patients with regard to frequency of each therapy. However, the frequency of treatments varied by age. In the oldest age group (median 80 years), about half as many patients (20.1%) were resected as in the youngest age group (median 54 years) (39.4%). Neoadjuvant, adjuvant, and palliative chemotherapy were also applied less frequently with increasing age, whereas the frequency of best supportive care increased with age (Table 1).

### 3.2 Survival Analysis

**FIGURE 1:**
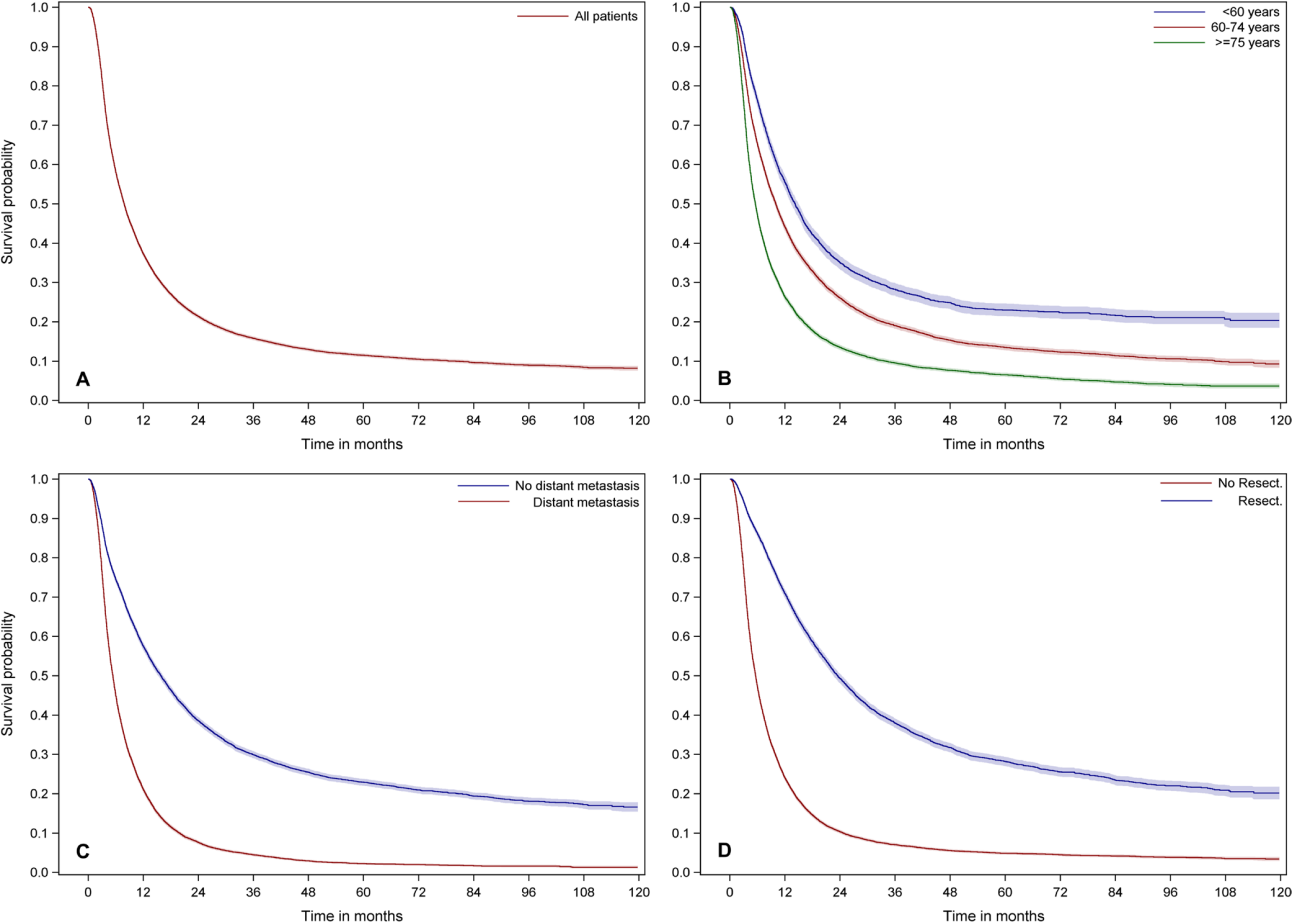
Survival of PDAC patients by age, stage and treatment. Kaplan–Meier curves show the overall survival (OS) of the total cohort of 23,339 patients with pancreatic ductal adenocarcinoma (PDAC) diagnosed from 2010 to 2017 (A) and subgroups according to age (B), stage (C) and resection status (D). There were significant differences in median OS according to age (< 60 years: 14.23 months (95% CI 13.38–14.98); 60–74 years: 10.03 months (95% CI 9.70–10.30); ≥ 75 years: 5.57 months (95% CI 5.41–5.70), *p* < 0.001), stage (localized: 15.87 months (95% CI 15.28–16.39); metastatic: 5.38 months (95% CI 5.25–5.48), *p* < 0.001) and resection status (resection: 23.51 months (95% CI 22.59–24.43)); no resection: (5.61 months (95% CI 5.51–5.70), *p* < 0.001).

The median overall survival (OS) of the whole study population (*n* = 23,339) was 7.84 months (95% CI (confidence interval) 7.67–8.00) (Figure 1). OS differed between age groups (< 60 years: 14.23 months (95% CI 13.38–14.98); 60–74 years: 10.03 months (95% CI 9.70–10.30); ≥ 75 years: 5.57 months (95% CI 5.41–5.70)), while there was no difference between female and male patients. Patients with localized disease had a three‐times longer median OS than patients with metastases (localized: 15.87 months (95% CI 15.28–16.39); metastatic: 5.38 months (95% CI 5.25–5.48)). The median OS of patients with resection (23.51 months (95% CI 22.59–24.43)) was approximately four times longer than that of patients without resection (5.61 months (95% CI 5.51–5.70)).

**FIGURE 2:**
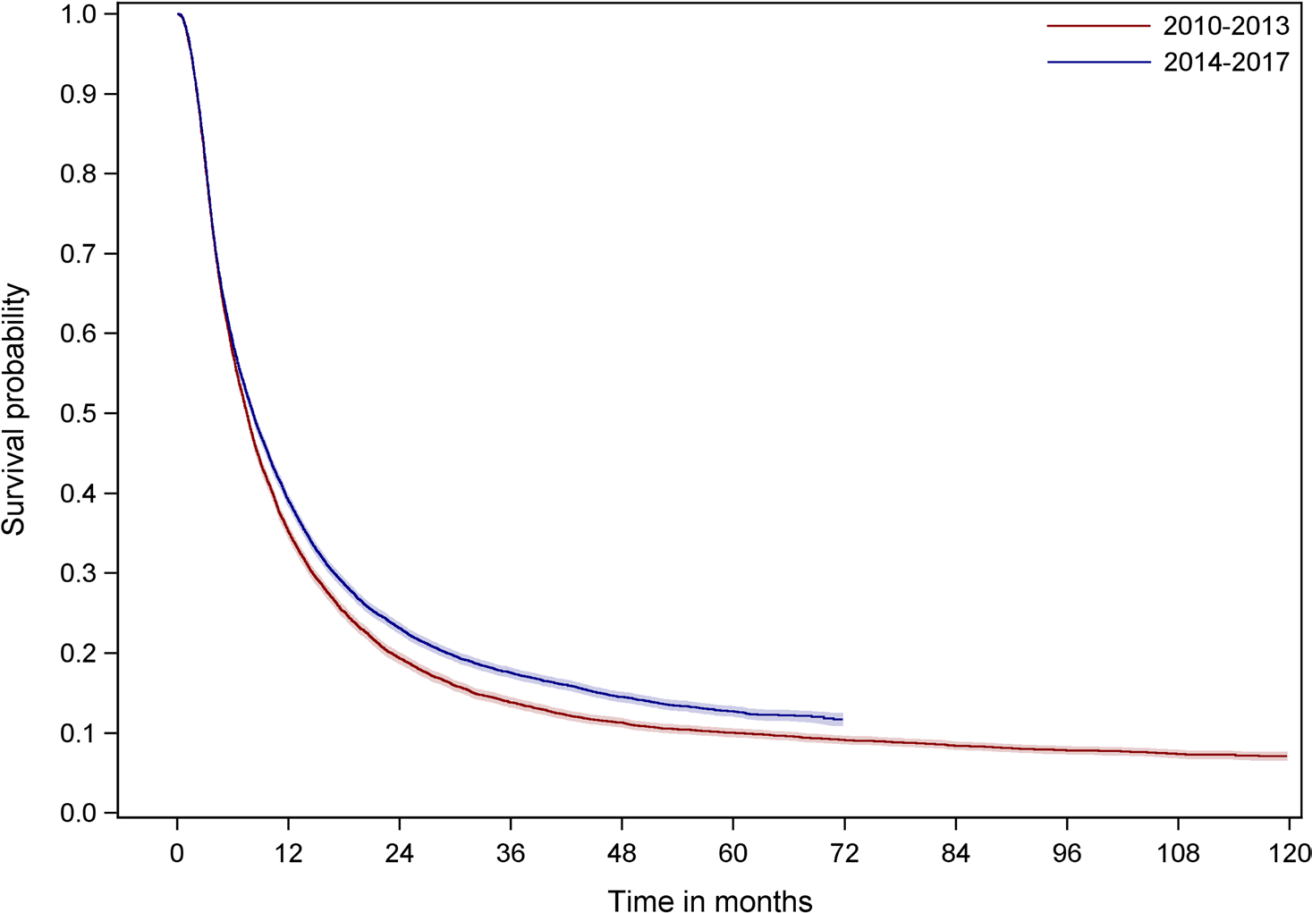
Survival of PDAC patients by year of diagnosis. Kaplan–Meier curves show the overall survival (OS) of 10,677 patients with pancreatic ductal adenocarcinoma (PDAC) diagnosed in the period from 2010 to 2013 and 12,662 patients with PDAC diagnosed in the period from 2014 to 2017. Log rank test showed a significant improvement of survival for patients diagnosed from 2014 to 2017 in comparison to patients diagnosed in the earlier period (2010–2014: 7.54 months (95% CI 7.31–7.70); 2014 to 2017: 8.20 months (7.97–8.43), *p* < 0.001). There were no significant differences of basic characteristics between the groups. The age‐, sex‐ and stage‐adjusted Cox regression showed a hazard ratio (HR) of 0.87 (95% CI 0.85–0.9; *p* < 0.001) for the diagnosis in the period from 2014 to 2017 compared to the earlier period (2010–2013).

Patients who were diagnosed in the period from 2014 to 2017 (8.20 months (7.97–8.43)) had a statistically significantly better prognosis than patients who were diagnosed in the earlier period from 2010 to 2013 (7.54 months (95% CI 7.31–7.70), *p* < 0.001) (Figure 2).

When comparing OS in both groups based on an age‐, sex‐, and stage‐adjusted Cox regression model, the hazard ratio (HR) was 0.87 (95% CI 0.85–0.9) for patients diagnosed from 2014 to 2017 as compared to those diagnosed earlier. Table 2 shows the characteristics of patients of the respective periods. There were no differences regarding demographics or tumor treatments between the groups.

**TABLE 2:** Characteristics of the subgroups by year of diagnosis.

	Year of diagnosis	
	2010–2013	2014–2017
Overall number^a^	10,677 (45.7%)	12,662 (54.3%)
Male	5004 (46.9%)	5896 (46.6%)
Female	5673 (53.1%)	6766 (53.4%)
Localized	4744 (44.4%)	5630 (44.5%)
Metastatic	5933 (55.6%)	7032 (55.5%)
Age at diagnosis (median)	73 (66–79)	74 (66–80)
Resection	2982 (27.9%)	3622 (28.6%)
Time to resection from diagnosis, days (median)	59 (36–83)	61 (36–84)
Total pancreatectomy^b^	343 (11.5%)	483 (13.3%)
Partial: distal^b^	527 (17.7%)	648 (17.9%)
Partial: Whipple^b^	2025 (67.9%)	2382 (65.8%)
Other^b^, ^c^	87 (2.9%)	109 (3%)
Explorative laparotomy, but no resection	614 (5.8%)	632 (5%)
Chemotherapy	5671 (53.1%)	6553 (51.8%)
Radiotherapy	458 (4.3%)	464 (3.7%)
Neoadjuvant therapy^d^	128 (3.6%)	214 (5.0%)
Adjuvant therapy^e^	1761 (59.1%)	2118 (58.5%)
Best supportive care^f^	3752 (35.1%)	4597 (36.3%)

*Note:* Shown are *n* (% of study population) or median (interquartile range).

^a^ Row percentages here referring to the entire study population (*n* = 23,339). In the remaining table, column percentages are shown.

^b^ % are based on all patients with resections in the respective cohort.

^c^ Unspecified partial resection of several segments of the pancreas.

^d^ Chemo‐ or radiotherapy within the period of PDAC diagnosis to resection or exploration. % are based on patients who were resected or had exploratory laparotomy without resection.

^e^ Chemo‐ or radiotherapy after curative resection within the 1st year after diagnosis. % are based on patients who were resected.

^f^ No tumor therapy (resection, chemo‐, or radiotherapy) in the 1st year after diagnosis.

Similarly favorable survival outcomes for the period from 2014 to 2017 compared to 2010 to 2013 were found in the subgroup of resected patients (HR 0.89 (0.87–0.92)) as well as in the group of non‐resected patients (HR 0.83 (0.78–0.88)) in the aforementioned adjusted models.

### 3.3 Trends in Therapy

**FIGURE 3:**
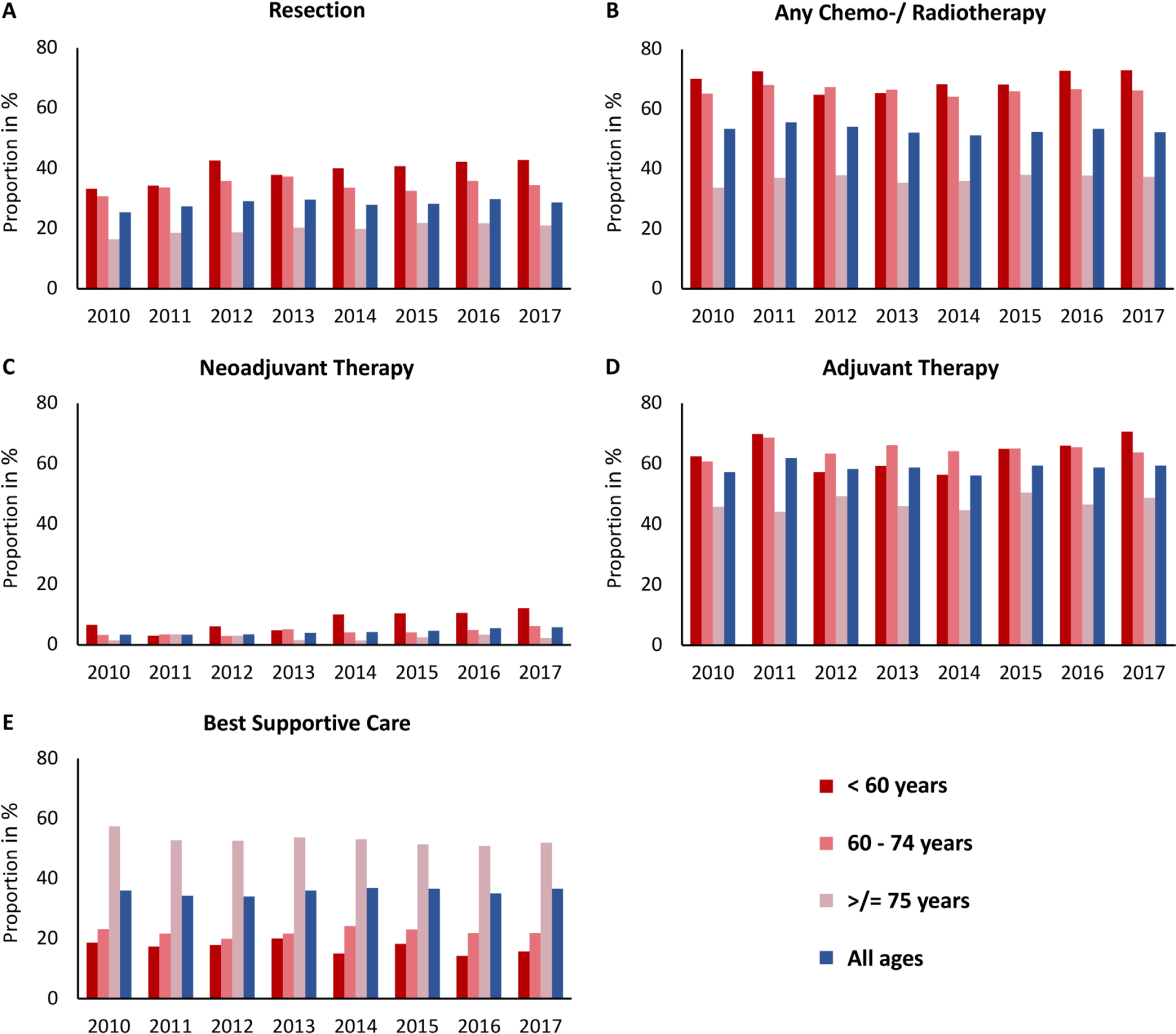
Trends in tumor therapies of 23,339 patients with pancreatic cancer from 2010 to 2017. The frequency of different tumor therapies in the first year after pancreatic cancer diagnosis of 23,339 patients, categorized according to different age groups, is shown. The *x*‐axis represents the corresponding years of diagnosis. Graph A and B correspond to the percentage of curatively resected patients (A) and patients who received any chemo‐ or radiotherapy (B) in the total study cohort. Neoadjuvant therapy (C) refers to the proportion of patients who received chemo‐ or radiotherapy prior to curative resection or surgical exploration without resection. Adjuvant therapy (D) includes all patients who received any chemo‐ or radiotherapy in the period following curative resection. Best supportive care (BSC) (E) corresponds to the proportion of the total cohort that were neither resected nor received chemo‐ or radiotherapy. Neoadjuvant therapy (C) increased steadily over the years in the overall cohort (*r* = 0.96, *p* < 0.001). This change was particularly noticeable in the young and middle‐aged groups (< 60 years: *R* = 0.81, *p* = 0.014; 60–74 years: *R* = 0.85, *p* = 0.008), but not in the older patients (≥ 75 years: *R* = 0.10, *p* = 0.80). There was also an increase in surgery (A) in the youngest (< 60 years: *R* = 0.80, *p* = 0.016) and the oldest age group (≥ 75 years: *R* = 0.90, *p* = 0.002). In the group of the oldest patients, BSC (E) was significantly declining over the years (≥ 75 years: *R* = −0.75, *p* = 0.031).

When comparing the frequency of different tumor therapies in the first year after PDAC diagnosis between 2010 and 2017, the most prominent change was seen in the use of neoadjuvant therapy (Figure 3). In relation to resected and explorative laparotomy patients, the proportion remained relatively low but increased steadily over the years in the overall cohort (*r* = 0.96) (Table 3). This change was particularly noticeable in the young and middle‐age groups (< 60 years: *r* = 0.81; 60–74 years: *r* = 0.85), but not in the group of the oldest patients (≥ 75 years: *r* = 0.10).

**TABLE 3:** Trends in tumor therapies from 2010 to 2017, stratified by age group.

Year	2010	2011	2012	2013	2014	2015	2016	2017	Pearson correlation	
Age	< 60 years								*r*	*p*
**All patients**	338	339	324	370	372	378	460	437		
**Resection**	112	116	138	140	149	154	194	187		
*% of all patients*	*33.1*	*34.2*	*42.6*	*37.8*	*40.1*	*40.7*	*42.2*	*42.8*	0.80	***0.016***
**Chemo‐ or radiotherapy**	237	246	210	242	254	258	335	319		
*% of all patients*	*70.1*	*72.6*	*64.8*	*65.4*	*68.3*	*68.3*	*72.8*	*73.0*	0.31	*0.456*
**Neoadjuvant therapy** ^a^	9	4	10	8	18	19	23	25		
*% of resection or expl. laparotomy*	*6.6*	*3.0*	*6.1*	*4.9*	*10.1*	*10.4*	*10.5*	*12.1*	0.85	***0.008***
**Adjuvant therapy** ^b^	70	81	79	83	84	100	128	132		
*% of resection*	*62.5*	*69.8*	*57.3*	*59.3*	*56.4*	*64.9*	*66.0*	*70.6*	0.31	*0.457*
**Best supportive care** ^c^	63	59	58	74	56	69	66	69		
*% of all patients*	*18.6*	*17.4*	*17.9*	*20.0*	*15.1*	*18.3*	*14.4*	*15.8*	*−0.59*	*0.125*

Age	60–74 years								r	p
**All patients**	1192	1169	1160	1206	1244	1205	1160	1120		
**Resection**	367	393	415	449	418	392	415	386		
*% of all patients*	30.8	33.6	35.8	37.2	33.6	32.5	35.8	34.5	0.33	*0.425*
**Chemo‐ or radiotherapy**	777	795	781	801	798	795	773	742		
*% of all patients*	65.2	68.0	67.3	66.4	64.2	66.0	66.6	66.3	*−0.14*	*0.743*
**Neoadjuvant therapy** ^a^	15	16	14	27	21	20	23	27		
*% of resection or expl. laparotomy*	3.2	3.4	2.9	5.1	4.2	4.1	4.9	6.2	0.81	***0.014***
**Adjuvant therapy** ^b^	223	270	263	297	268	255	272	246		
*% of resection*	60.8	68.7	63.4	66.2	64.1	65.1	65.5	63.7	0.10	*0.813*
**Best supportive care** ^c^	276	254	231	262	301	278	254	245		
*% of all patients*	23.2	21.7	19.9	21.7	24.2	23.1	21.9	21.9	0.09	*0.838*

Age	≥ 75 years								*r*	*p*
**All patients**	999	1101	1160	1319	1474	1571	1566	1675		
**Resection**	164	204	217	267	293	343	340	351		
*% of all patients*	16.4	18.5	18.7	20.2	19.9	21.8	21.7	21.0	0.90	***0.002***
**Chemo‐ or radiotherapy**	337	408	440	467	531	597	593	626		
*% of all patients*	33.7	37.1	37.9	35.4	36.0	38.0	37.9	37.4	0.58	*0.128*
**Neoadjuvant therapy** ^a^	3	9	8	5	5	10	14	9		
*% of resection or expl. laparotomy*	1.5	3.5	3.0	1.5	1.4	2.5	3.4	2.2	0.10	*0.808*
**Adjuvant therapy** ^b^	75	90	107	123	131	173	158	171		
*% of resection*	45.7	44.1	49.3	46.1	44.7	50.4	46.5	48.7	0.44	*0.271*
**Best supportive care** ^c^	574	581	611	709	783	808	798	870		
*% of all patients*	57.5	52.8	52.7	53.8	53.1	51.4	51.0	51.9	−0.75	***0.031***

Age	All ages								*r*	*p*
**All patients**	2529	2609	2644	2895	3090	3154	3186	3232		
**Resection**	643	713	770	856	860	889	949	924		
*% of all patients*	25.4	27.3	29.1	29.6	27.8	28.2	29.8	28.6	0.62	*0.103*
**Chemo‐ or radiotherapy**	1351	1449	1431	1510	1583	1650	1701	1687		
*% of all patients*	53.4	55.5	54.1	52.2	51.2	52.3	53.4	52.2	−0.55	*0.158*
**Neoadjuvant therapy** ^a^	27	29	32	40	44	49	60	61		
*% of resection or expl. laparotomy*	3.4	3.4	3.5	3.9	4.3	4.6	5.5	5.8	0.96	***< 0.001***
**Adjuvant therapy** ^b^	368	441	449	503	483	528	558	549		
*% of resection*	57.2	61.9	58.3	58.8	56.2	59.4	58.8	59.4	0.01	*0.978*
**Best supportive care** ^c^	913	894	900	1045	1140	1155	1118	1184		
*% of all patients*	36.1	34.3	34.0	36.1	36.9	36.6	35.1	36.6	0.43	*0.287*

*Note:* Italicized: Rows with percentages. Italicized bold: *p* values < 0.05.

Abbreviation: Expl. laparotomy, explorative laparotomy.

^a^ Chemo‐ or radiotherapy within the period of pancreatic cancer diagnosis to resection or exploration.

^b^ Chemo‐ or radiotherapy after curative resection within the 1st year after diagnosis.

^c^ No tumor therapy (resection, chemo‐, or radiotherapy) in the 1st year after diagnosis.

There was also an increase in the proportion of the resected patients in the youngest (< 60 years: *r* = 0.80) and the oldest age group (≥ 75 years: *r* = 0.90) over time. Of note, in the group of the oldest patients, the proportion without tumor therapy was declining (≥ 75 years: *r* = −0.75). The proportion with any chemotherapy (i.e., neoadjuvant, adjuvant and/or palliative) or radiotherapy, as well as the proportion with adjuvant therapy, showed no relevant change over the study period.

The tables of Tables S3 and S4 show the frequency of different tumor therapies in the first year after PDAC diagnosis between 2010 and 2017 separately for patients with localized and metastatic stage. Overall, there were more significant changes in treatment frequency over time in patients with localized disease (Table S3) than in patients with metastases (Table S4). In addition, the changes regarding the increasing frequency of resections and neoadjuvant therapy, such as a decreasing frequency of best supportive care in this cohort, mainly affected the groups of the youngest (< 60 years) and oldest patients (≥ 75 years).

## 4 Discussion

Our study, which is the largest study in the German‐speaking area and one of the largest studies in Europe, provides comprehensive insights into changes in the treatment of pancreatic cancer and survival over time. This real‐world analysis demonstrates that overall survival of PDAC is still very poor but improving. In the most recent period, an increasing proportion of patients received anticancer therapies, while a decreasing proportion with best supportive care was documented. Novel treatment concepts, such as neoadjuvant therapy, were increasingly being implemented in daily practice.

### 4.1 Study Population

The distribution of age and stage of the study cohort was comparable to cancer registry data from Germany, which indicates a high degree of representativity [3]. As seen in German cancer registry data, the present study also shows the presence of metastatic disease in > 50% of cases at diagnosis, although there was no access to the exact TNM tumor classification of the patients. In addition, a study from the Dutch Cancer Registry including > 36,000 PDAC patients diagnosed between 1997 and 2016 showed a distribution of age and stage comparable to our data [11]. This stage distribution was unchanged in the periods from 2010 to 2013 and 2014 to 2017, indicating that diagnosis in earlier stages, for example, through more precise diagnostics, did not occur.

### 4.2 Survival

In line with our data, several real‐world studies showed that overall survival in pancreatic cancer unfortunately is still very limited and differs significantly from those in randomized controlled trials (RCT) in which patients with relevant comorbidities, advanced age, or poor performance status were usually excluded [7, 14, 15, 16].

The median OS of patients after resection was 23.5 months (95% CI 22.6–24.4) in the present analysis, which is considerably lower than the 54.4 months after resection and adjuvant mFOLFIRINOX or 35.0 months with adjuvant gemcitabine in a RCT published in 2018 [7]. However, in the previous ESPAC‐4 and ESPAC‐3 studies, the median OS was less favorable for the same regime (25.5 and 23.6 months for gemcitabine) [17, 18]. These differences could be explained by different patient characteristics of the individual studies, selection of patients by strict inclusion criteria, but also by general improvements in medical care. However, comparable real‐world data from Europe are rare. For example, median OS after resection in the Netherlands from 2013 to 2016 was 18.1 months and thus about 5 months shorter than in the present study [11]. Population‐based data from the Denmark Cancer Registry (2011–2018) show a slightly longer median OS (25.8 months) compared to the present study [10].

The same pattern is seen in metastatic patients. While RCTs showed a median OS of 6.7–11.1 months, the current study found a median OS of 5.4 months (95% CI 5.3–5.5) [14, 15, 16]. However, comparability is considerably difficult as the patients included in the aforementioned RCTs were on average approximately 10 years younger than those in the current study. This demonstrates that RCTs do not cover the full patient population that needs to be addressed in clinical practice. Also, real‐world data from the Dutch Cancer Registry showing a median OS for this patient population (5.9 months) similar to our study, with up to 79% receiving no treatment, underline the difference between RCTs and the real‐world setting [11].

Despite the worse prognosis compared to RCTs, we see a statistically significant improvement in survival over time. However, the difference is marginal, i.e., the median increase in mOS for the entire study population is only about 3 weeks when comparing the periods of 2010 to 2014 (mOS 7.5 months (95% CI 7.3–7.7)) with 2014–2017 (8.2 months (95% CI 8.0–8.4)).

Similar trends have been described for other European countries as well as for non‐European countries. There was an improvement in median OS from 3.4 months (2004–2010) to 5.0 months (2011–2019) in Denmark and from 3.1 months (1997–2000) to 3.8 months (2013–2016) in the Netherlands, although the survival rates were considerably lower compared to the present study [10, 11]. In Victoria, Australia, median OS improved from 2.7 months (metastatic) and 13.3 months (nonmetastatic) in 2011 to 3.9 months and 15.9 months in 2015, respectively [19].

Causes of these improvements are likely multifactorial. Increased centralization and standardization of care and improved oncologic and surgical treatment options are being discussed. For instance, a recent analysis of German health claims data showed an increase in the proportion of patients receiving initial treatment in certified centers for several oncologic diseases from 2009 to 2017, including pancreatic cancer. PDAC patients who received initial treatment in a certified center had an estimated mean survival advantage of 2 months compared to those who were treated in a noncertified center [20]. Furthermore, Dutch Cancer Registry data on resected patients showed an increase in center‐based treatment (> 40 resections/year), an increase in neoadjuvant treatment approaches, and an increase in minimally invasive surgery between 2011 and 2019. However, these developments were not associated with a higher R0 resection rate, which is a key predictor of survival. In fact, the R0 resection rate even decreased due to increasing numbers of comprehensive pathological assessments [21].

### 4.3 Trends in Therapy

Treating a larger proportion of patients by reducing the morbidity of various surgical and oncologic therapies may be critical to the improvement of survival. In the present analysis, the number of resected patients increased by approximately one‐third from 33% in 2010 to 43% in 2017 in the youngest group (< 60 years) and from 16% to 21% in the oldest group (=/> 75 years). There were also increases from 8% to 17% in the Netherlands and Denmark, although the rates were significantly lower than in the present study of German health claims data with 29% in all patients diagnosed in 2017 [10, 11]. A large population‐based study of several European cancer registries and the SEER database (U.S.) including > 140,000 patients with PDAC also showed relevant differences in resection rates between countries ranging from 13% to 22% in the years 2003–2014 [22].

Why resection rates in Germany are significantly higher than in the countries mentioned cannot be conclusively determined. Population‐based comparative data for verification are currently not available. However, similarly high resection rates have already been described, e.g., in Victoria, Australia, with 31% in the period 2011–2015 [19]. There were also marked differences between countries in chemotherapy and radiotherapy rates, which show that the international standardization of PDAC therapies should be further expanded [22].

While other studies have reported an increase in the rate of chemotherapy, we observed this only for neoadjuvant but not for adjuvant or palliative therapy [10, 11, 22, 23, 24]. The present analysis shows that the rate of neoadjuvant therapy was still relatively low but almost doubled in the youngest (< 60 years) and middle‐aged (60–75 years) groups from 7% to 12% and from 3% to 6%, respectively. One advantage of neoadjuvant therapy is better tolerability than that of adjuvant therapy. Chemotherapy delivery is most likely the most important nonsurgical factor to improve survival [25]. However, in upfront resectable PDAC, there are concerns about progression of PDAC during neoadjuvant therapy, which eventually makes a cure impossible. While neoadjuvant therapy is currently considered standard of care for borderline resectable or locally advanced pancreatic cancer, the benefit for resectable patients is controversial [26]. The NEONAX study did not show a significantly better OS for Gemcitabine and Nab‐Paclitaxel perioperatively versus upfront surgery and adjuvant Gemcitabine and Nab‐Paclitaxel [25]. Similar results were obtained by the NORPACT‐1 study, where mFOLFIRINOX upfront did not reveal a survival benefit [27].

A large analysis from several countries in North and South America showed also an increasing rate of neoadjuvant therapy and a better 30‐day survival of those patients [28]. Patients receiving neoadjuvant therapy were more often white, were treated in teaching hospitals, were privately insured, and younger.

In general, the increase in the frequency of tumor therapies in PDAC patients is described in most studies, but a causal relationship to the improvement in median OS cannot be drawn.

### 4.4 Limitations

Some limitations of the study must be taken into account when interpreting the results. First, information on stage is not available in detail in health claims data, but there are codes for affected lymph node involvement and metastases. While metastatic status has been shown to be plausibly coded in claims data, affected lymph node status tends to be underrecorded in this data source and was therefore not analyzed as a separate category [13]. In addition, information on other important prognostic factors such as site of the metastatic disease (e.g., liver, lungs etc.) and pathology results with histological or molecular subtypes was not available. Second, we used data from four health insurances, i.e., did not have full population coverage and excluded patients whose continuous insurance was interrupted. However, comparisons with cancer registry data did not indicate that our patient population lacked representativeness. Third, the fact that outpatient diagnoses were only available on a quarterly basis may account for the relatively high rate of pancreatic resections in metastatic patients that do not meet the standard of care. Apart from a few exceptions, early metastatic recurrences were likely coded in the same period as the resection. This most likely resulted in a falsely high rate of resections in patients with metastatic disease. It has to be noted, however, that the distinction between de novo metastatic disease and early metastatic recurrence is also suboptimal in cancer registry data. Fourth, it was not possible to clearly differentiate between adjuvant and palliative chemotherapy because disease recurrence was not captured in the available data. Intended neoadjuvant therapy was also not possible to differentiate from adjuvant or palliative therapy if no resection was performed, except for the cases receiving explorative laparotomy.

Nevertheless, the strengths of the study, such as the size of the population, the real‐world approach, and the first comprehensive insight into changes in pancreatic cancer therapies and survival over time, should be emphasized.

## 5 Conclusion

The present analysis shows a reasonable trend toward more tumor therapy and less best supportive care, although only a minimal survival benefit could be demonstrated. The wide international variation in the frequency of different therapies highlights the need for further standardization. It should be pointed out that, compared to other countries, there is a particularly high rate of resections in the population studied, although the causes and risk–benefit considerations remain to be investigated in further studies.

## Author Contributions

**Marko Damm:** conceptualization (lead), formal analysis (equal), writing – original draft (lead), writing – review and editing (equal), visualization (equal), project administration (lead). **Miriam Heinig:** conceptualization (equal), methodology (equal), formal analysis (equal), data curation (lead), writing – original draft (equal), writing – review and editing (lead), visualization (equal). **Jonas Rosendahl:** supervision (supporting). **Patrick Michl:** supervision (supporting). **Ulrike Haug:** conceptualization (supporting), methodology (equal), writing – review and editing (equal), supervision (lead). **Sebastian Krug:** conceptualization (supporting), writing – original draft (equal), writing – review and editing (equal), supervision (equal). All authors have read and agreed to the published version of the manuscript.

## Ethics Statement

We used the German Pharmacoepidemiological Research Database (GePaRD) which is based on claims data from four statutory health insurance providers in Germany. In Germany, the utilization of health insurance data for scientific research is regulated by the Code of Social Law, specifically §75 Book X. All involved health insurance providers as well as the German Federal (Social) Insurance Office and the Senator for Science, Health, and Consumer Protection in Bremen as their responsible authorities approved the use of GePaRD data for this study. According to the Ethics Committee of the University of Bremen studies based on GePaRD are exempt from institutional review board review.

## Consent

Informed consent for studies based on GePaRD is required by law unless obtaining consent appears unacceptable and would bias results, which was the case in this study.

## Conflicts of Interest

The authors declare no conflicts of interest.

## Supporting information


**Data S1:** Supporting Information.

## Data Availability

Data can be requested from the corresponding author in compliance with applicable data protection regulations.
