# Supplementary material for: Trends in the Treatment and Survival of Pancreatic Cancer: Analysis of 23,339 Patients Diagnosed Between 2010 and 2017
Source: Cancer Med. 2025 Sep 19;14(18):e71248. doi: 10.1002/cam4.71248 (PMC12447353; doi:10.1002/cam4.71248)
Supplement: Supplementary file 1 — Data S1: Supporting Information. [file CAM4-14-e71248-s001.docx]

**Damm et al.**

**Trends in the treatment and survival of pancreatic cancer:**

**Analysis of 23,339 patients diagnosed between 2010 and 2017**

# Supplemental Material

**Supplement 1:**

**Identification of incident pancreatic cancer patients in GePaRD**

To identify incident pancreatic cancer patients in GePaRD, i.e. German health claims data, we used an algorithm which we developed based on case reviews. In a first step, all patients were selected who had at least one in- or outpatient diagnosis code of pancreatic cancer (ICD-10-GM “C25”) and a continuous health insurance period of at least two years before the first in- or outpatient pancreatic cancer diagnosis code was recorded. Patients with at least one “status post” diagnosis in the two years before the first pancreatic cancer diagnosis were excluded. Second, patients were excluded if an inpatient discharge diagnosis of another cancer that had likely metastasized to the pancreas (malignant neoplasms of lung, skin, or kidney, i.e. ICD-10-GM “C34”, “C43” or “C64”) was recorded within three months before or after the first inpatient pancreatic cancer diagnosis. Among remaining patients, we considered those with at least one inpatient discharge diagnosis of pancreatic cancer as incident pancreatic cancer cases. Patients without an inpatient discharge diagnosis but outpatient diagnosis codes for pancreatic cancer were only considered as incident pancreatic cancer cases if further criteria were fulfilled (e.g. pancreatic diagnostics, indicators of end-stage disease). The date of the first in- or outpatient pancreatic cancer diagnosis code was assigned as the date of incident diagnosis. We compared the incidence determined based on this algorithm in GePaRD with cancer registry data and found very good agreement.

Because our analyses focused on pancreatic ductal adenocarcinoma, incident pancreatic cancer patients as identified above were excluded if an in- or outpatient diagnosis code indicating neuroendocrine pancreatic cancer (ICD-10-GM “C25.4”) was recorded within three months after the incident pancreatic cancer diagnosis, or if a treatment indicating neuroendocrine pancreatic cancer (Interferon-alpha, Dacarbazine, Diazoxide, Octreotide, Lanreotide, Everolimus, Streptozotocin, Temozolomide, Sunitinib, Doxorubicin, or Bevacizumab) was recorded in the in- or outpatient setting ever after the incident pancreatic cancer diagnosis.

**Supplement 2:**

**Characteristics of PDAC patients excluded due to interruption of continuous insurance (N=2606)**

|  | **All** |
| --- | --- |
|  | **2,606 (100%)** |
| **Available follow-up (years)** | 1.3 (0.8-2.0) |
| **Year of diagnosis** |  |
| 2010-2013 | 989 (38.0%) |
| 2014-2017 | 1,617 (62.0%) |
| **Sex** |  |
| Male | 1,585 (60.8%) |
| Female | 1,021 (39.2%) |
| **Age at diagnosis** | 71 (61-76) |
| **Age group** |  |
| <60 years | 531 (20.4%) |
| 60-74 years | 1,208 (46.4%) |
| >/=75 years | 867 (33.3%) |
| **Stage** |  |
| Localized | 972 (37.3%) |
| Metastatic | 1,634 (62.7%) |

Shown are n (% of excluded patients) or median (Interquartile range).

**Supplement 3:**

**Trends in therapy of 10,374 patients with localized pancreatic cancer from 2010 to 2017**

|  |  |  |  |  |  |  |  |  |  |  |
| --- | --- | --- | --- | --- | --- | --- | --- | --- | --- | --- |
| **Year** | **2010** | **2011** | **2012** | **2013** | **2014** | **2015** | **2016** | **2017** | **Pearson correlation** | |
| **Age** | **<60 years** | | | | | | | | **r** | ***p*** |
| Resection (% of all patients) | 60.1 | 66.2 | 68.8 | 65.7 | 73.0 | 68.1 | 69.5 | 70.9 | 0.73 | ***0.041*** |
| Chemo-/ radiotherapy (% of all patients) | 56.5 | 56.6 | 46.1 | 55.1 | 52.8 | 60.4 | 64.8 | 64.5 | 0.65 | *0.083* |
| Neoadjuvant therapy^1^  (% of resection or expl. laparotomy) | 3.2 | 2.1 | 3.4 | 2.4 | 4.8 | 7.5 | 7.1 | 10.0 | 0.90 | ***0.003*** |
| Adjuvant therapy^2^ (% of resection) | 61.4 | 65.6 | 50.0 | 56.4 | 52.9 | 62.1 | 63.7 | 66.0 | 0.27 | *0.518* |
| Best supportive care^3^ (% of all patients) | 21.0 | 21.3 | 20.8 | 16.9 | 14.1 | 14.3 | 11.4 | 13.3 | -0.93 | ***0.001*** |
| **Age** | **60-74 years** | | | | | | | | **r** | ***p*** |
| Resection (% of all patients) | 57.4 | 64.8 | 67.5 | 66.2 | 60.3 | 62.1 | 68.4 | 64.2 | 0.34 | *0.409* |
| Chemo-/ radiotherapy (% of all patients) | 54.7 | 63.8 | 60.6 | 61.7 | 56.9 | 59.1 | 60.6 | 56.5 | -0.12 | *0.770* |
| Neoadjuvant therapy^1^  (% of resection or expl. laparotomy) | 1.9 | 3.0 | 3.0 | 3.1 | 2.7 | 3.1 | 5.4 | 4.4 | 0.79 | ***0.018*** |
| Adjuvant therapy^2^ (% of resection) | 56.3 | 67.4 | 61.0 | 64.0 | 61.3 | 61.5 | 62.5 | 60.7 | 0.04 | *0.919* |
| Best supportive care^3^ (% of all patients) | 21.0 | 15.3 | 13.9 | 15.6 | 20.3 | 17.4 | 15.6 | 19.5 | 0.07 | *0.871* |
| **Age** | **>/=75 years** | | | | | | | | **r** | ***p*** |
| Resection (% of all patients) | 28.6 | 31.8 | 30.7 | 36.3 | 35.7 | 37.1 | 39.0 | 36.9 | 0.90 | ***0.003*** |
| Chemo-/ radiotherapy (% of all patients) | 28.2 | 29.0 | 30.9 | 31.8 | 28.5 | 29.8 | 32.0 | 30.4 | 0.47 | *0.235* |
| Neoadjuvant therapy^1^  (% of resection or expl. laparotomy) | 2.0 | 4.0 | 2.2 | 1.6 | 0.4 | 1.7 | 2.3 | 1.0 | -0.50 | *0.207* |
| Adjuvant therapy^2^ (% of resection) | 44.6 | 41.2 | 45.2 | 47.5 | 42.8 | 46.9 | 45.5 | 45.1 | 0.36 | *0.381* |
| Best supportive care^3^ (% of all patients) | 56.2 | 52.6 | 52.5 | 49.3 | 51.2 | 50.9 | 47.2 | 49.5 | -0.83 | ***0.011*** |
| **Age** | **all ages** | | | | | | | | **r** | ***p*** |
| Resection (% of all patients) | 45.6 | 49.7 | 50.9 | 52.4 | 49.8 | 49.6 | 53.6 | 50.8 | 0.61 | *0.110* |
| Chemo-/ radiotherapy (% of all patients) | 43.8 | 46.8 | 45.2 | 47.1 | 42.6 | 43.7 | 46.7 | 43.9 | -0.15 | *0.730* |
| Neoadjuvant therapy^1^  (% of resection or expl. laparotomy) | 2.2 | 3.2 | 2.9 | 2.5 | 2.3 | 3.3 | 4.6 | 4.2 | 0.74 | ***0.035*** |
| Adjuvant therapy^2^ (% of resection) | 54.1 | 59.3 | 54.8 | 57.5 | 53.4 | 55.8 | 56.5 | 55.8 | -0.05 | *0.905* |
| Best supportive care^3^ (% of all patients) | 35.8 | 33.3 | 32.4 | 31.2 | 34.7 | 34.8 | 31.0 | 34.4 | -0.17 | *0.679* |

| ^1^ Chemo- or radiotherapy within the period of PDAC diagnosis to resection or exploration. |
| --- |
| ^2^ Chemo- or radiotherapy after curative resection within the 1st year after diagnosis. |
| ^3^ No tumor therapy (resection, chemo-, or radiotherapy) in the 1st year after diagnosis.  Abbreviations: Expl. laparotomy: Explorative laparotomy |

**Supplement 4:**

**Trends in therapy of 12,965 patients with metastatic pancreatic cancer from 2010 - 2017**

| **Year** | **2010** | **2011** | **2012** | **2013** | **2014** | **2015** | **2016** | **2017** | **Pearson correlation** | |
| --- | --- | --- | --- | --- | --- | --- | --- | --- | --- | --- |
| **Age** | **<60 years** | | | | | | | | **r** | ***p*** |
| Resection (% of all patients) | 14.5 | 12.8 | 18.8 | 12.0 | 14.4 | 15.3 | 19.2 | 18.4 | 0.53 | *0.176* |
| Chemo-/ radiotherapy (% of all patients) | 79.5 | 83.3 | 81.8 | 75.0 | 80.4 | 75.5 | 79.6 | 80.3 | -0.26 | *0.528* |
| Neoadjuvant therapy^1^  (% of resection or expl. laparotomy) | 14.3 | 5.1 | 12.8 | 12.5 | 22.6 | 18.4 | 18.8 | 17.5 | 0.64 | *0.084* |
| Adjuvant therapy^2^ (% of resection) | 65.5 | 84.6 | 81.3 | 73.9 | 70.0 | 76.7 | 72.9 | 86.0 | 0.27 | *0.511* |
| Best supportive care^3^ (% of all patients) | 17.0 | 14.8 | 15.3 | 22.9 | 15.8 | 21.9 | 16.8 | 17.9 | 0.28 | *0.495* |
| **Age** | **60-74 years** | | | | | | | | **r** | ***p*** |
| Resection (% of all patients) | 12.5 | 11.7 | 11.9 | 14.3 | 12.3 | 13.2 | 12.2 | 13.2 | 0.32 | *0.439* |
| Chemo-/ radiotherapy (% of all patients) | 72.4 | 71.0 | 72.4 | 70.1 | 69.9 | 70.5 | 71.0 | 73.2 | 0.00 | *0.997* |
| Neoadjuvant therapy^1^  (% of resection or expl. laparotomy) | 5.8 | 4.6 | 2.4 | 10.8 | 7.9 | 6.1 | 3.5 | 10.7 | 0.35 | *0.402* |
| Adjuvant therapy^2^ (% of resection) | 75.0 | 73.8 | 73.4 | 74.0 | 75.3 | 76.0 | 78.0 | 74.4 | 0.51 | *0.192* |
| Best supportive care^3^ (% of all patients) | 24.6 | 26.2 | 24.5 | 26.6 | 27.3 | 26.8 | 26.4 | 23.6 | 0.03 | *0.948* |
| **Age** | **>/=75 years** | | | | | | | | **r** | ***p*** |
| Resection (% of all patients) | 6.2 | 6.0 | 8.0 | 6.7 | 6.3 | 8.0 | 7.2 | 8.2 | 0.63 | *0.096* |
| Chemo-/ radiotherapy (% of all patients) | 38.3 | 44.6 | 44.2 | 38.4 | 42.5 | 45.5 | 42.8 | 42.9 | 0.34 | *0.414* |
| Neoadjuvant therapy^1^  (% of resection or expl. laparotomy) | 0.0 | 1.7 | 4.9 | 1.3 | 4.3 | 4.8 | 6.7 | 5.3 | 0.81 | ***0.016*** |
| Adjuvant therapy^2^ (% of resection) | 50.0 | 58.8 | 63.3 | 39.6 | 54.0 | 65.2 | 50.8 | 61.8 | 0.21 | *0.610* |
| Best supportive care^3^ (% of all patients) | 58.5 | 52.9 | 52.9 | 57.5 | 54.7 | 51.9 | 54.1 | 53.9 | -0.40 | *0.328* |
| **Age** | **all ages** | | | | | | | | **r** | ***p*** |
| Resection (% of all patients) | 10.4 | 9.6 | 11.1 | 10.6 | 9.7 | 11.0 | 10.8 | 11.3 | 0.52 | *0.187* |
| Chemo-/ radiotherapy (% of all patients) | 60.6 | 62.4 | 61.5 | 56.4 | 58.4 | 59.2 | 58.7 | 58.6 | -0.56 | *0.153* |
| Neoadjuvant therapy^1^  (% of resection or expl. laparotomy) | 6.0 | 3.9 | 5.2 | 8.1 | 9.5 | 7.6 | 8.1 | 9.9 | 0.79 | ***0.019*** |
| Adjuvant therapy^2^ (% of resection) | 67.5 | 72.1 | 71.9 | 64.1 | 67.9 | 72.4 | 68.1 | 72.2 | 0.17 | *0.695* |
| Best supportive care^3^ (% of all patients) | 36.3 | 35.0 | 35.4 | 40.2 | 38.7 | 38.1 | 38.4 | 38.4 | 0.62 | *0.105* |

| ^1^ Chemo- or radiotherapy within the period of PDAC diagnosis to resection or exploration. |
| --- |
| ^2^ Chemo- or radiotherapy after curative resection within the 1st year after diagnosis. |
| ^3^ No tumor therapy (resection, chemo-, or radiotherapy) in the 1st year after diagnosis. |
| Abbreviations: Expl. laparotomy: Explorative laparotomy |
